# Trans-papillary bilio-pancreatic stenting: When how and which stent

**DOI:** 10.3389/fgstr.2022.1092263

**Published:** 2023-01-05

**Authors:** Annalisa Cappello, Rosario Landi, Christian Gerges, Vincenzo Cennamo, Guido Costamagna, Andrea Tringali

**Affiliations:** ^1^ Gastroenterology and Interventional Endoscopy, Azienda Unità Sanitaria Locale di Bologna (AUSL Bologna), Bologna, Italy; ^2^ Digestive Endoscopy Unit, Fondazione Policlinico Universitario Agostino Gemelli IRCCS, Rome, Italy; ^3^ Centre for Endoscopic Research Therapeutics and Training (CERTT), Università Cattolica del Sacro Cuore, Rome, Italy; ^4^ Digestive Endoscopy, Evangelisches Krankenhaus, Düsseldorf, Germany; ^5^ Department of General Surgery, Gastroenterology and Interventional Endoscopy, Azienda Unità Sanitaria Locale di Bologna (AUSL Bologna), Bologna, Italy

**Keywords:** endoscopic retrograd colangiopancreatography, stent, malignant biliary stenosis, benign biliary stenosis, chronic panceatitis, post ERCP pancreatitis, transpapillary stenting, SEMS (self-expandable metallic stent)

## Abstract

Nowadays, stenting malignant biliary stenosis (extrahepatic or hilar), benign biliary stenosis, and pancreatic duct stenosis in chronic pancreatitis as well as stenting for prophylaxis of post- endoscopic retrograde cholangiopancreatography pancreatitis and for failed extraction of biliary stones or endoscopic papillectomy are the many common challenges for a bilio-pancreatic endoscopist. The purpose of this review is to provide a practical approach to bilio-pancreatic stenting indications and techniques. Having a thorough understanding of stenting indications and techniques, for a bilio-pancreatic endoscopist means being able to develop a tailored approach for each clinical scenario depending on the type of stent used. Biliary stents, in fact, vary in diameter, length, and composition, making it possible to give each patient personalized treatment.

## 1 Introduction

The bilio-pancreatic stenting performed to drain the malignant stenoses of the common bile duct, has several indications for malignant and benign diseases involving the common bile duct, the hepatic hilum, and the pancreatic duct in the present day.

A bilio-pancreatic endoscopist must have a thorough understanding of stenting indications and techniques to develop a tailored approach for each clinical scenario. The choice regarding the type of stent to be used is an important step. Biliary stents vary in diameter, length, and composition, making it possible to give each patient personalized treatment.

The purpose of this review is to provide a practical approach to bilio-pancreatic stenting indications and techniques.

## 2 Malignant extrahepatic biliary stenosis

Malignant stenoses of the extrahepatic biliary tract are most frequently caused by pancreatic tumors, predominantly located at the level of the head or uncinate process, and by cholangiocarcinoma. Other causes of malignant stenosis of the extrahepatic biliary tract include ampullary/duodenal tumors, gallbladder tumors, and metastases of other tumors infiltrating the head of the pancreas and the main biliary duct (1).

Before endoscopic drainage, the diagnosis and stage of the disease must be established (2, 3) by cross-sectional imaging and tissue acquisition.

### 2.1 Preoperative drainage

Preoperative drainage should not be routinely performed. In fact, early surgery leads to fewer complications than preoperative biliary drainage (4, 5). Preoperative drainage should be reserved for patients with cholangitis, severe jaundice, itching, patients who are candidates for neoadjuvant therapy, or patients whose surgery is expected to be delayed by more than 2 weeks (2).

If a decision is made to place a preoperative biliary drain, the endoscopic route should be preferred over the percutaneous route (6) because percutaneous drainage, in addition to having higher morbidity (7), may lead to the development of tumor seeding (8) thus compromising the curability of the disease.

Regarding the type of stent to be used, Self-Expandable Metal Stents (SEMS) have a lower obstruction rate than Plastic Stents (PS) (9–11) and maintain patency longer (12).

In addition, it has been seen that, in patients who need to undergo neoadjuvant therapy, the use of SEMS compared to the use of PS leads to a lower incidence of delay in the performance of neoadjuvant therapy while maintaining similar overall treatment costs (13). The cost-effectiveness analysis by Almadi et al. (14) also shows that placement of SEMS compared with PS is not only cost-effective but also associated with a higher likelihood of achieving the oncologic outcomes needed to perform surgery sooner. In the case of preoperative drain placement, therefore, the use of 10-mm SEMS is recommended (2).

**Figure 1:**
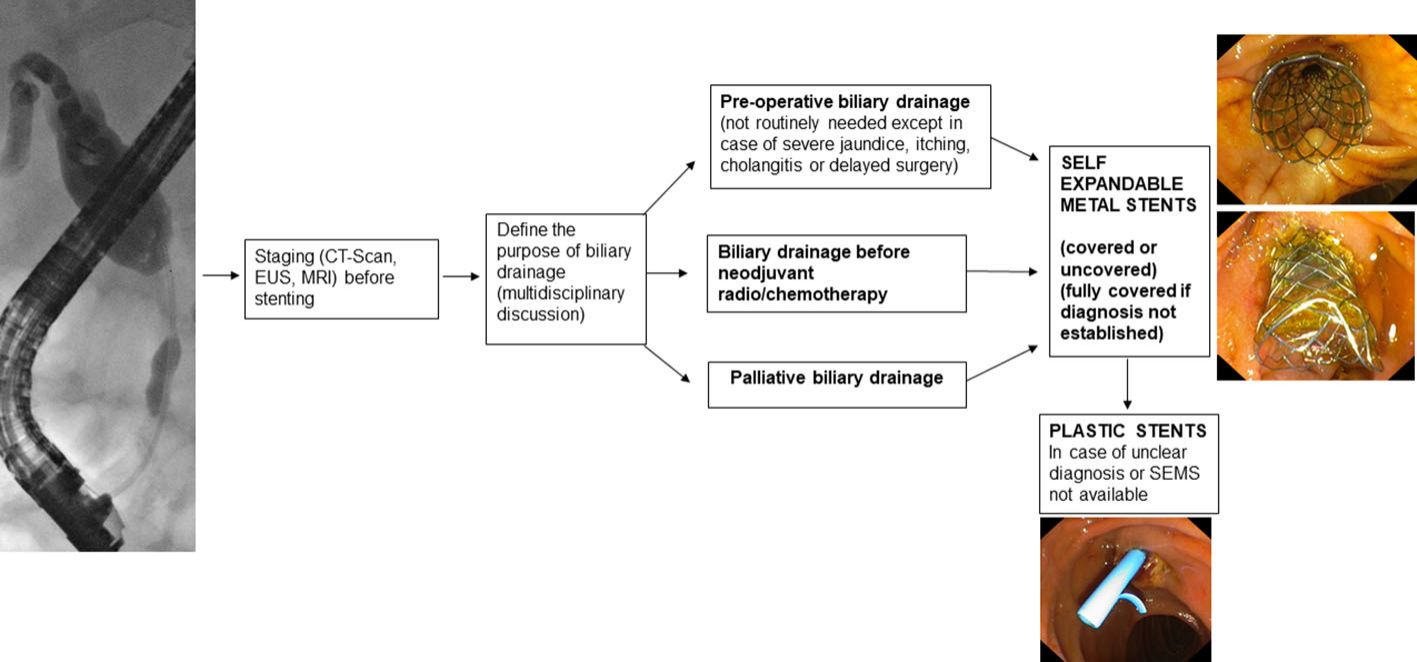
Malignant bile duct strictures and choice of the stent.

Regarding the type of SEMS, Fully Covered Self-Expandable Metal Stents (FCSEMS) show a lower complication rate than PS (15, 16). Acute pancreatitis occurred more frequently when the FCSEMS is place but it does not affect the waiting time for surgery (15). The FCSEMS should be preferred if the diagnosis is not histologically confirmed (**Figure 1**).

In patients with biliary obstruction due to pancreatic adenocarcinoma who are candidates for neoadjuvant therapy, several randomized controlled trials (RCTs) have been performed: in the RCT conducted by Gardner et al. (17), in which the use of FCSEMS, Uncovered Self-Expandable Metal Stents (USEMS), and PS were compared, FCSEMS were seen to lead to a shorter delay in the performance of neoadjuvant therapy. In the RCT conducted by Seo et al. (18), FCSEMS and USEMS were shown to maintain equal drainage function in the two groups (72.2% vs 72.9%). The reasons for stent failure were mainly tumor ingrowth for USEMS and stent migration for FCSEMS. The incidence of cholecystitis was similar in the two groups. However, in a recent meta-analysis (19), including 2358 patients, the FCSEMS were superior to the USEMS with respect to prevent recurrent biliary obstruction. Although there was no significant difference in total procedure-related adverse events between the two types of SEMS.

### 2.2 Palloatove drainage

In reference to palliative drainage of malignant stenosis of the extrahepatic biliary tract, the transpapillary endoscopic approach with placement of SEMS is recommended (2, 20). In RCTs (21–24) performed on patients with unresectable distal malignant biliary obstruction, SEMS placement, as compared to PS, demonstrated a higher patency rate, lower incidence of complications, and greater cost-effectiveness. Even in patients with fewer than 3 months of survival and with metastatic disease in whom SEMS or PS had been placed, costs were similar (25) and better quality of life was shown in SEMS patients at follow-up (26). The most recent meta-analysis available in the literature on the drainage of unresectable distal biliary duct malignant stenosis (27), confirmed a higher patency rate with SEMS, a lower incidence of stent dysfunction, and a lower incidence of requiring reintervention in SEMS-bearing patients compared to PS-bearing patients. In addition, in the subgroup analysis, the study showed that placement of Partially Covered Self-Expandable Metal Stents (PCSEMS) or FCSEMS compared with PS placement leads to increased mean survival.

Nevertheless, the choice of SEMS type remains controversial because both USEMS and FCSEMS placement have advantages and disadvantages. The most recent meta-analysis (28) confirms that in patients with FCSEMS versus USEMS there is no difference in the incidence of stent failure, mortality, or development of complications. Likewise, in the RCT by Conio et al. (29), there was no significant difference in the incidence of stent malfunction or survival of patients with FCSEMS or USEMS. The causes of FCSEMS malfunction are stent migration (7% vs 0% in the USEMS group) and stent obstruction by sludge (8% in the FCSEMS group) or tumor overgrowth (4% in the FCSEMS group), while the leading cause of USEMS malfunction is tumor ingrowth (10% in the USEMS group). However, in cases where the diagnosis is not definite, a USEMS should not be placed because tissue ingrowth reduces its long-term patency and compromises its possibility of removal, which, even using the “stent-in-stent” technique (30, 31) may be difficult (32) or impossible.

In a recent RCT (33) the use of USEMS was compared with the use of PCSEMS and showed a higher incidence (p=0.0467) of occlusion in USEMS (43.8%) compared with PCSEMS (22.7%) with a significantly longer mean stent patency (p=0.0112) in the PCSEMS-carrying group (455 days) compared with the USEMS-carrying group (301 days).

Regarding the incidence of cholecystitis, an increased risk of cholecystitis in patients carrying FCSEMS was not found in the literature (34–37). However, in some studies (38), in patients with gallbladder *in situ*, the proximal end of the FCSEMS was placed below the orifice of the cystic duct. Risk factors for the development of cholecystitis (39, 40) appear to be tumor involvement in the cystic duct, the presence of cholelithiasis, and opacification with contrast medium of the gallbladder before stent placement.

In single stent placement, there is no need for routine sphincterotomy if PCSEMS or USEMS are placed. The RCT conducted by Hayashi et al. (41) on 200 patients with distal malignant stenosis from unresectable pancreatic cancer showed that performing sphincterotomy before PCSEMS placement does not lead to a reduction in the risk of post-endoscopic retrograde cholangiopancreatography (ERCP) pancreatitis and other adverse events. The most recent meta-analysis available in the literature (42) indeed shows that performing a sphincterotomy before placement of a stent, leads to an increased incidence of the risk of bleeding and cholangitis, and the risk of developing post-procedure pancreatitis is unchanged. Regarding the placement of FCSEMS, it remains controversial whether a sphincterotomy should be performed because there is a hypothetical risk that FCSEMS will close the pancreatic duct outlet, increasing the risk of post-ERCP pancreatitis (2).

In the case of malfunctioning SEMS placed in patients with distal malignant biliary stenosis, a PS or new SEMS should be placed inside the previous SEMS (2, 43). One possible advantage of FCSEMS is that covered stents can be removed and subsequent replaced with a new SEMS (43).

Several new types of stents are in development, with the aim of prolonging the function of FCSEMS. For example, FCSEMS with a chemotherapeutic agent incorporated into the stent covering material, have been tested on animals and small cohorts of patients with malignant stenosis of the distal biliary duct. To date, available studies do not show an advantage in terms of clinical outcomes over standard SEMS placement (44, 45). FCSEMS with antireflux valves (46–48) and FCSEMS with anti-migration systems (49, 50) have also been studied with promising results. Studies with larger patient samples and longer-term follow-ups are needed to validate the present results.

## 3 Malignant hilar biliary stenosis

Malignant hilar biliary stenoses can occur by intraductal development of primary biliary tract tumors or local extension of tumors, such as gallbladder cancer, or by ab extrinsic compression in the case of metastatic lymph node involvement at the hilar level (51). The indications for endoscopic stent placement to drain malignant hilar biliary stenoses are preoperative drainage and palliation.

Before intervention to decompress the biliary tract, the patient should be studied using all necessary imaging techniques, because the accuracy of Computed Tomography (CT) and Magnetic resonance imaging (MRI) disease staging decreases after biliary stent placement due to ductal decompression and imaging artifacts (2, 52). Magnetic resonance cholangiopancreatography (MRCP) can provide a detailed “road-map” to perform optimal biliary drainage avoiding the scenario of “opacified and undrained bile ducts” and thus reducing the rate of infectious complications. In addition, imaging techniques such as CT/MRI, in cases of portal vein thrombosis, can assess the presence and extent of hepatic atrophy. Drainage of an atrophic liver segment should be avoided because it increases the risk of developing cholangitis (53) and does not lead to increased clinical success and survival (54).

The approach to drain malignant hilar stenosis should be established by a multidisciplinary team in high-volume referral centers and each case should be considered unique (55). In the multidisciplinary setting, the actual malignancy of the stenosis, its extent, and the most appropriate therapeutic approach should be ascertained to achieve adequate drainage of intrahepatic biliary branches of functioning liver parenchyma (56). Suboptimal drainage of intrahepatic bile ducts is the main reason for the high rate of cholangitis secondary to drainage of malignant hilar stenosis (57) and can turn a patient with a potentially resectable tumor into an inoperable one.

Bismuth and Corlette’s classification (58) divides malignant hilar stenosis into 4 types based on the involvement of the main hepatic duct and central segmental ducts. Depending on the type of stenosis, the number of stents to be inserted can be roughly estimated. Type I is a non-hilar stenosis located in the proximal hepatic duct more than 2 cm below the hilar confluence so placement of a single stent can ensure complete drainage. In type II, the confluence of the main hepatic ducts is involved, so two stents are needed to drain the left and right hemisystems. In type IIIa and IIIb, the confluence and the right (anterior/posterior) or left (IV/II and III segments) secondary ducts are involved; in this case, 3 stents would be needed to drain all the intrahepatic branches. Type IV reflects an advanced disease state in which both left and right main and secondary ducts are involved and theoretically 4 stents would be needed.

Because hilar stenoses are often complex, as in type IIIa for example, to achieve complete drainage, it must not only be decided whether a unilateral or bilateral stent should be placed but the need for multisectoral stent placement must be considered as well (59).

### 3.1 Preoperative drainage

The indication and type of preoperative biliary drainage should be decided by a multidisciplinary team based on patient characteristics and institutional experience (2).

Placement of a preoperative biliary drain is associated with an increased risk of postoperative infection (60, 61), and in some surgeries, such as left hepatectomy, it is generally not indicated (61, 62). However, there are conditions associated with a high risk of postoperative liver failure, such as an estimated hepatic residual volume ≤ 30% after surgery, for which preoperative drain placement is indicated (63).

In patients who are candidates for major hepatic resection with bilirubinemia > 3 mg/dL, the preoperative biliary drainage, limited to the future liver remnant has been suggested.

Regarding the choice between endoscopic and percutaneous approaches for placement of a preoperative biliary drain, ASGE guidelines (64) suggest the first-line use of endoscopic drainage over percutaneous drainage based on the evidence of a higher incidence of tumor metastasis in patients treated with percutaneous drainage than those treated with endoscopic drainage (65, 66).

Generally, percutaneous drainage is preferred when the patient has altered gastro-duodenal anatomy, when the bile ducts to be drained are not accessible with ERCP, and when adequate endoscopic biliary drainage has not been obtained.

If preoperative endoscopic drainage of hepatic hilum stenosis is performed, plastic stents (PS) or nasobiliary tubes are indicated (67).

Although less comfortable for the patient, nasobiliary drainage is found to lead to a lower incidence of cholangitis, postoperative fistulas, and stent malfunction (68). In fact, a lower incidence of occlusion with the nasobiliary tube than with the PS has been reported (69).

PS placement is indicated preoperatively and when a decision on the type of treatment, curative or palliative, has not yet been established. They are preferred in these cases because they are easily removable and do not prevent further therapeutic procedures. In addition, their diameter and length can be adapted to the intrahepatic biliary tree, and when sufficient drainage is not achieved, multiple PSs can be inserted to improve bile outflow.

PS insertion in malignant hilar stenoses should be performed by (1) selective cannulation with a guide wire of the intrahepatic ducts upstream of the hilar stenosis; (2) selective anterograde injection of contrast into the selected bile duct to define the anatomy of the stenosis; (3) balloon dilation (4-6 mm) of the stenosis, if necessary; (4) insertion of plastic stents to drain the opacified duct, thus avoiding the scenario of “injected and undrained ducts”.

### 3.2 Palliative drainage

The goals of palliative biliary drainage are used to relieve jaundice and prevent cholangitis improving quality of life and allowing possible radiation and chemotherapy treatments (70).

Guidelines (2, 64) recommend that the choice of drainage type should be determined based on stenosis characteristics, local expertise, and patient preference. Bismuth type I and II stenoses should first be addressed endoscopically whereas, with the Bismuth type III and IV stenoses an endoscopic, percutaneous, or combined approach can be used, depending on the type of stenosis. In fact, in cases of complex hilar stenosis, the placement of a percutaneous drain and an endoscopic drain should not be considered exclusive of each other. For example, it could be initially placed a percutaneous drain, which could then be used as a guide to place a stent endoscopically (Rendez-vous technique) (71).

Regardless of the technique used, biliary tract drainage should involve at least 50% of the liver volume reducing the risk of infection and liver failure. The minimum portion of the liver volume, excluding tumor volume, that should be drained has been analysed in several studies (53, 72, 73) which conclude that drainage of more than 50 percent of the liver volume is an independent factor for a greater decrease in bilirubin levels, a lower incidence of cholangitis, and a longer survival rate.

In the case of established malignancy and palliative purpose of hilar stenosis drainage, USEMS are recommended because they allow, compared with other types of SEMS, drainage of the lateral bile ducts through the uncovered meshes (74). In addition, compared with PSs, USEMS improve survival and ensure longer-lasting drainage patency (75). While USEMS have a higher cost than PS, they also present potential cost savings due to the higher patency rate leading to the requirement for fewer re-interventions (76, 77). However, it must be kept in mind that once placed, the USEMS are difficult or even impossible to remove. This may jeopardize possible subsequent surgical treatments so the USEMS should only be placed in cases of established palliative treatment of malignant hilar stenosis. Furthermore, regarding the placement of the USEMS, adequate stent release must be studied because inadequate stent placement can lead to significant difficulties in subsequent reinterventions when placing additional drains between the stent meshes. Therefore, in cases where there is uncertainty about the diagnosis and/or inability to obtain adequate endoscopic drainage, PS placement is recommended (64).

Placement of USEMS should be done only after an adequate cannulation of intrahepatic ducts. To avoid contrast opacification of ducts that cannot be subsequently drained, opacification with contrast medium should be avoided before performing guidewire cannulation of intrahepatic ducts (78–80). In this technique, once the stenosis is crossed with a guidewire, contrast medium is injected before stent insertion. Some authors have also proposed not using contrast medium at this stage either, keeping the narrowing of the stent at the level of the stenosis as the only reference point for stent placement during the release of the proximal end of the stent (81).

When more than one stent needs to be placed, guidewires should be placed first, possibly several, over all the stenoses and they should be dilated to 6 mm to facilitate subsequent stent placement. It is usually preferred to place the stent in the left hepatic duct first, which is generally more angled. In case of difficulties in placing the stent due to the stenosis being very angulated, it is suggested to switch from a “floppy” guidewire to a more rigid one.

The choice of USEMS depends on the ductal diameter, the number of USEMS required, and the length of the stenosis. There are different USEMS available on the market today (82). Their length varies from 4 to 12 cm and their diameter from 6 to 10 mm. Commonly for hilar strictures 8-10 cm long and 8-10 mm diameter USEMS are needed.

However, placement of multiple USEMS to drain intrahepatic ducts remains a complex procedure since it is often difficult to advance other metallic stents into the biliary duct after the first metallic stent has been released. In case of difficulties, a stiffer guidewire can be used to change the insertion angle of the metallic stent to be inserted, alternatively the technique described by Hookey et al. (83) can be used, in which an 8.5 F straight plastic stent, with the proximal flange removed, is placed at the level of the main biliary duct to facilitate the passage of a second metal stent after the release of a first metal stent.

An alternative technique to the placement of multiple side-by-side USEMS (SBS) is the stent-in-stent (SIS) placement technique, also known as Y-stent. In this technique, one stent is released, often into the left hepatic duct for greater angulation, and a second stent is then released into the other hepatic duct through the meshes of the first stent. The SIS technique is most widely used in Japan and Korea (84) because it does not expose the main biliary duct to over-dilatation. Many USEMS dedicated to the SIS approach have been developed: in most of these, only the central part of the stent can be used to insert another stent between the meshes while in some of these, the uniform mesh size allows the insertion of another stent in each region of the USEMS (85, 86). The SBS technique is preferred in the West because, in the event of stent obstruction, endoscopic reintervention is usually successful, unlike the SIS technique where reintervention may be prohibitive (87).

The distal margin of the USEMS in the SIS configuration should be placed at the transpapillary (88),”shotgun” level to facilitate possible future reintervention in case of stent failure. However, some authors disagree and believe that avoiding transpapillary placement could potentially keep Oddi’s sphincter intact leading to a possible reduction in the risk of developing cholangitis (89).

After the USEMS release, the main problem that can develop in the long term is stent occlusion. Stent occlusion can occur by sludge deposition or by tissue ingrowth and/or overgrowth. Sludge can be removed with a Fogarty balloon, while tissue ingrowth and/or overgrowth can be resolved by inserting a second USEMS or a plastic stent, depending on life expectancy.

The patency of USEMS can be increased by applying photodynamic therapy (90) or radiofrequency ablation (91, 92), prior to stent insertion. These techniques seem promising, but they are not widely used, are expensive and data to date are limited (93).

## 4 Benign biliary stenosis

Benign biliary tract stenoses can occur post-surgically or be correlated to bilio-pancreatic diseases. Chronic pancreatitis is the disease that most frequently leads to benign biliary tract stenosis. Post-surgical stenoses, on the other hand, are mainly associated with laparoscopic cholecystectomy surgeries and liver transplant surgeries (94).

While in malignant stenoses endoscopic treatment is limited to providing drainage of the biliary tract, in benign stenoses endoscopic treatment with stent placement is used for therapeutic purposes and is directed at the regression of the stenosis. After stent placement in malignant biliary stenoses, an endoscopic re-intervention is performed only on demand, whereas in benign biliary stenoses the scheduling of subsequent endoscopic follow-up appointments for removal/replacement of placed stents is recommended (2). In this regard, in the endoscopic treatment of benign biliary stenoses, a sphincterotomy is recommended to facilitate retreatment with repeated stent replacements.

Endoscopy is the first-line treatment in the treatment of benign biliary stenoses (95) and involves the placement of PS or FCSEMS that are selected according to the type of stenosis to be approached (2).

If a PS is used, placement of multiple parallel plastic stents (MPS) is recommended (96), inserting as many stents as possible every 3-4 months for 1 year (97). Recent studies propose prolonging plastic stent replacement to every 6 months in selected categories of patients (98, 99).

To insert MPS first it is often necessary to dilate the stenosis at the first treatment with a 4-6 mm balloon depending on the size of the bile duct upstream of the stenosis. After insertion of the first PS, the bile duct is again cannulated with a 6F catheter on a hydrophilic guidewire and another stent is placed parallel to the previous one. The procedure is repeated until the maximum number of stents (preferably 10F in diameter) are placed, according to the degree of stenosis and the diameter of the biliary duct upstream and downstream of the stenosis. During the procedure, good coordination between operator and assistant is required to avoid intrahepatic migration of the stents (100).

In contrast, in the case of FCSEMS placement, it is generally not necessary to perform dilatation of the stenosis before stent insertion. However, when releasing FCSEMS it should be considered that some FCSEMS shorten significantly after release and that the distal part of the FCSEMS should be placed 5-10 mm downstream of the papilla to facilitate its future removal. Replacement of FCSEMS is recommended every 6 months to prevent degradation of the membrane covering the metal mesh of the stent, although there is little evidence for this (101, 102).

A recent meta-analysis comparing the use of MPS with the use of FCSEMS in benign biliary stenosis (103) found no differences in the incidence of stenosis resolution, stenosis recurrence, and adverse events in the two groups. Another meta-analysis (104) reported that a significantly lower number of procedures in the FCSEMS group was required. Nevertheless, long-time follow-up is available for MPS only.

FCSEMS have the advantage of having a longer patency than MPS (105) but have a migration risk of about 9%, which varies according to the etiology of the stenosis on which the stent was applied (106). Indeed, it has been seen that in patients with chronic pancreatitis, migration of FCSEMS occurs less frequently than in biliary tract stenosis with other etiology [4.6% versus 14% (p = 0.006)] (107). To reduce the risk of FCSEMS migration, a double pigtail plastic stent can be placed within the FCSEMS (108).

Placement of FCSEMS also presents the risk of stenosis development at the proximal margin of the stent especially if the diameter of the placed stent is larger than the diameter of the biliary duct upstream of the stenosis (109). Therefore, it seems appropriate to consider using FCSEMS with a diameter of 8 mm rather than the more commonly used 10 mm.

It is crucial to select the right stent on a case-by-case basis depending on the length of the stenosis, its location, and its etiology (102).

### 4.1 Benign biliary tract stenoses related to chronic pancreatitis

Biliary tract stenoses from chronic pancreatitis may have different etiology: they may be secondary to scarring of the pancreatic parenchyma following repeated inflammatory episodes (110), secondary to acute inflammation of the pancreatic parenchyma, or secondary to compression of pancreatic pseudocysts (111). In the latter two conditions, obstruction resolves with resolution of the acute inflammatory process or with drainage of the pseudocyst.

Stenoses of the distal biliary duct secondary to a scarring process of the pancreatic parenchyma, especially if pancreatic calcifications are present (112), tend to be refractory to endoscopic treatment more than other benign stenoses of the biliary tract (113) because they have a greater extension and are located at the level of the calcific fibrotic tissue of the head of the pancreas.

Before determining the treatment of biliary stenosis secondary to chronic pancreatitis, it is mandatory to exclude the presence of a tumor with cross-sectional imaging and echoendoscopy. In cases of benign scarring stenosis of the biliary duct, endoscopic treatment represents the first line of treatment, being less invasive than surgical treatment (95), and is indicated in symptomatic patients and in cases of persistent elevation of alkaline phosphatase and/or bilirubin values for more than one month (114).

Endoscopic treatment of scarring stenosis of the biliary duct from chronic pancreatitis involves the temporary insertion of MPS or FCSEMS (114).

The use of MPS involves cannulating the biliary duct, performing sphincterotomy, dilating the stenosis, and inserting multiple 10 F PSs. The procedure should be repeated every 3-4 months until the incision of the stenosis is no longer visible on a balloon cholangiography and the contrast medium flows freely into the duodenum. Placement of MPS has demonstrated clinical success ranging from 44% to 92% (97).

Treatment with FCSEMS, on the other hand, involves the insertion of an FCSEMS at the level of the distal biliary duct and generally does not require endoscopic reintervention before 6 months. FCSEMS are often preferred because they have a larger diameter than multiple plastic stents (a 10 mm FCSEMS has the equivalent diameter of 7 10 F PSs) and require less endoscopic reintervention (115).

Two RCTs (116, 117), performed in patients with benign distal stenosis of the biliary duct secondary to chronic pancreatitis, demonstrated equal clinical success and equal adverse events in patients treated with MPS and patients treated with FCSEMS. The RCT conducted by Ramchandani et al. (117) with a 2-year follow-up, showed 77.1% (54/70) stenosis resolution in the group with MPS and 75.8% (47/62) in the group with FCSEMS. The group of patients with MPS was treated with placement of at least two 8.5 or 10 F PS side-by-side. Patients were then endoscopically re-evaluated at 4 and 8 months for replacement and addition of more PSs. At 12 months, the PSs were removed, and the therapy outcome was evaluated using cholangiography. In the group of patients carrying FCSEMS, an 8- or 10-mm diameter FCSEMS was placed, and the endoscopic removal procedure was scheduled after 12 months in which, as in the other group, the outcome of treatment was also evaluated with cholangiography. This study brought an important technical contribution: a reasonable safety profile was demonstrated in the removal of in the removal of FCSEMS at 12 months, rather than at 6 months as in other studies (118). Based on this evidence, a cost-effective analysis was recently conducted that demonstrated that treatment with FCSEMS with stent removal after 12 months is cost-effective compared with treatment with MPS (119). However, further studies with more prolonged follow-up are needed because there may also be recurrences of late stenosis (120).

### 4.2 Benign biliary tract stenosis post liver transplantation

Post-liver transplantation biliary tract stenoses arise more frequently after living donor transplantation than after deceased donor transplantation (121) and can be distinguished into anastomotic biliary stenosis (ABS) and non-anastomotic biliary stenosis (NABS) (122).

ABS are the most common and can occur at both duct-to-duct biliary anastomoses and hepaticojejunal anastomoses. They are usually single, short stenoses and are located, in the case of deceased donor transplantation, at the level of the middle choledochus or, in the case of living donor transplantation, near the hepatic hilum. ABS are secondary to the fibrotic healing process of the biliary epithelium and local tissue ischemia (123).

NABS, on the other hand, are localized intra- and extrahepatically at least 5 mm proximal to the anastomosis. Their etiopathogenesis is multifactorial (124) but is generally associated with underlying tissue ischemia (125).

A peculiar feature of post-transplant stenoses is that, even in cases of tight stenosis, the bile ducts upstream of the stenosis, probably due to the presence of fibrosis, do not show the same degree of dilatation as would occur in non-transplanted liver ducts (126).

Persistent elevation of cholestasis indices after transplantation, even in asymptomatic patients, should raise suspicion of biliary stenosis (127, 128). Suspected biliary stenosis should therefore be investigated by inserting contrast medium into the T-tube if still in place, or if not, by an MRCP scan (129).

The therapeutic approach to post-transplant stenosis should be discussed in multidisciplinary settings in highly specialized centers (130).

For stenoses of duct-to-duct anastomoses the first-line approach is endoscopic, whereas for stenoses on hepaticojejunal anastomoses the percutaneous approach is generally preferred. Although, with the improvement of enteroscopic techniques (131, 132) endoscopic access to the papilla is now possible in 68-93% of cases (133, 134).

Endoscopic treatment usually involves performing sphincterotomy, balloon dilatation (4-6 mm) of the stenosis, and placement of MPS. Because of the small diameter of the donor ducts, it is sometimes necessary to insert small caliber MPS (7 or 8.5 F). Every 3 months the procedure is repeated with additional stents added until the stenosis is resolved (135). The response time to endoscopic therapy varies according to the time of presentation of stenosis after the liver transplantation. In fact, ABS arising in the first 30 days postoperatively are associated with a good response within 6 months of treatment (136), while late presentation ABS (>90 days) generally need prolonged treatment to avoid recurrence (137).

In the recent prospective study by Tarantino et al. (138) of 87 patients with post-transplant ABS stenosis, treatment with MPS demonstrated 98.9% clinical success in an average treatment time of 8 months with an average of 4.7 endoscopic procedures and an average placement of 3.7 PS.

However, treated patients need to be followed up over time because recurrence of stenosis can occur even years after treatment. In fact, long-term follow-up of MPS-treated patients demonstrated recurrence of stenosis in about 6 percent of patients with an average follow-up of 5.8 years (139). In the case of recurrence of stenosis, endoscopic treatment with dilatation and placement of MPS is effective (140).

To prevent the need to repeat endoscopic procedures every 3 months to replace and add PS, the use of FCSEMS was also studied in patients with ABS. The use of FCSEMS would allow stent replacement every 6 months resulting in only two endoscopic procedures per year. Three RCTs were performed (141–143) that did not demonstrate superiority of treatment with FCSEMS over treatment with MPS. The high migration rate is the main limiting factor for the use of FCSEMS in ABS (144). A recent systematic review of studies of endoscopically treated ABS patients reported a FCSEMS migration rate of 16% (145). To reduce the risk of FCSEMS migration, an FCSEMS with an antimigration waist can be used that can be fully released intraductally due to the presence of a long wire that reaches the duodenum and allows easy subsequent removal. This stent has demonstrated a migration rate reduced to 0-3% (146, 147). FCSEMS with antimigration fins have also been developed and have shown promising results (145).

Treatment of NABS is more complicated, especially if stenoses arise more than 1 year after transplantation (148), and long-term therapeutic success is limited to 50-75% of cases with up to 25-50% of patients eventually requiring retransplantation or dying (149).

### 4.3 Post-cholecystectomy biliary tract injuries

Post-laparoscopic cholecystectomy biliary tract injuries are usually the result of direct surgical trauma and may be related to a leak from the cystic stump, lesions of the main biliary duct, and/or lesions of the aberrant right sectoral hepatic ducts.

The clinical presentation varies depending on the type of lesion found and whether a leak is present or not. The MRCP is important in planning the therapeutic strategy (150).

For postoperative stenosis of the main biliary duct, the endoscopic approach is the first line of treatment and involves MPS insertion with reintervention every 3-4 months, increasing the number of stents until the resolution of the stenosis has been achieved (151). FCSEMS stents may be considered if the stenosis is located at least 2 cm below the hepatic confluence (2).

After treatment of stenosis with MPS, patients with an average follow-up of 13 years had no recurrence of stenosis in 88.6% (31/35) of cases, and the recurrences were all successfully treated endoscopically (152).

A recent cohort study (153) of 154 patients with post-cholecystectomy biliary tract stenosis treated with MPS reported a resolution of stenosis in 96.7% of patients. Patients underwent an average of 4.2 ± 1.5 endoscopic procedures over an average treatment period of 11.8 ± 6.4 months with the placement of an average number of 4.3 ± 1.6 stents.

The use of FCSEMS in post-cholecystectomy stenosis was recently evaluated in a subgroup analysis of an international study on the evaluation of the efficacy of FCSEMS in benign biliary stenosis (154). It showed significantly inferior results compared to treatment with MPS. In fact, after a mean permanence time of 10.9 months, the resolution of stenosis occurred in only 72% (13/18) of patients and at 5-year follow-up, only 61% of these patients had no recurrence of stenosis.

In the case of leakage from the cystic duct or the Luschka’s duct, the goal is to eliminate the transpapillary pressure gradient to better drain the bile. This can be achieved by placing a PS in the main biliary duct with or without the performance of sphincterotomy (155). Two recent meta-analyses (156, 157) compared the treatment of biliary duct leaks with sphincterotomy alone, with PS placement alone, or with PS placement associated with the performance of sphincterotomy. They revealed that combined treatment is associated with a higher therapeutic success rate than the other treatments.

In case of refractory leaks, MPS or FCSEMS placement should be a strategy for rescue endotherapy (158).

Regarding aberrant segmental bile duct lesions (159), if an aberrant duct stenosis is found, treatment with MPS is effective. However, for the remaining types of damage, the indication and outcome of endoscopic treatment vary depending on the type of lesion reported according to Strasberg’s classification (160). In fact, for lesions in which there is complete transection of the aberrant duct with a leak, cannulation of the aberrant duct from the cystic stump or the main biliary duct may be attempted. If successful, a plastic stent may be placed to create an “endoscopic anastomosis” and thus achieve closure of the fistula. After 4-8 weeks after placement of the plastic stent, removal of the stent and subsequent dilatation of the aberrant duct with the placement of MPS can be performed. In contrast, in the case of complete occlusion of the aberrant duct without a leak, the “wait and see” strategy is preferred considering the high endoscopic failure rate and possible good outcome without any specific treatment.

## 5 Pancreatic duct stenosis in chronic pancreatitis

The main symptom of chronic pancreatitis is pain. Pain is exacerbated by alcohol intake, by cigarette smoking and by a diet inadequate for pancreatic function. Ductal hypertension, a condition that develops as a result of the presence of pancreatolithiasis and/or pancreatic duct stenosis, plays a major role in its etiopathogenesis.

The treatment of a patient with chronic pancreatitis with a pancreatic duct stenosis who presents with pain symptoms unresponsive to medical therapy should be discussed in a multidisciplinary setting. The patient should be studied by imaging techniques, such as CT and MR with MRCP, to rule out an underlying malignancy. MRCP is the most accurate imaging technique to visualize pancreatic duct stenosis and assess the extent of dilatation upstream of the duct (161).

Guidelines (114, 162) recommend as the first-line endoscopic treatment of pancreatic stenosis, the insertion at the level of the stenosis of a single 10 F straight plastic stent, which can be left in place for 1 year if pain symptoms are in remission. The endoscopic procedure involves cannulation of the main pancreatic duct, sphincterotomy, pneumatic dilatation of the stenosis, and subsequent placement of a plastic stent of 8.5 or 10 F with a variable length, depending on the size of the stenosis. The procedure leads to pain remission in 70-94% of patients (163).

However, about one-third of patients require prolonged stenting beyond 1 year (164). In this group of patients, more aggressive treatment may improve the clinical outcome, which is why the guidelines suggest considering MPS insertion increasing the radial force of dilatation. Treatment with MPS in patients with refractory pancreatic stenosis was proposed in 2006 by Costamagna et al. (165) who reported the treatment of 19 patients in whom an average of 3 8.5-11.5 F PSs were placed and were removed after 6-12 months. This treatment resulted in the resolution of pain symptoms in 84% of patients and no major complications were reported. Prior to MPS placement, the endoscopic procedure involves removal of the previously placed single pancreatic plastic stent, cannulation with a 0.035-inch hydrophilic guidewire of the pancreatic duct, and subsequent pneumatic dilation of the stenosis with a dilation balloon selected according to the diameter (6-10 mm) and length (2 ± 4 cm) of the stenosis. In the placement of multiple PSs, the stent of the largest extension and size (10 or 11.5 F) is inserted first to reduce the risk of stent migration into the pancreatic duct during the release of subsequent PSs. After the first stent, as many stents of the same or smaller size as possible are inserted into the stenosis. After 6 to 12 months of treatment, all PSs are removed endoscopically with forceps or a loop, dilatation of the stenosis is verified by passing a 12 mm balloon, and, upon completion, a 6 F nasopancreatic drainage tube is placed with the tip in the tail of the pancreas. Temporary placement of a nasopancreatic drainage tube allows the outflow of contrast medium from the pancreatic duct into the duodenum to be checked and any onset of pain during forced injection to be noted.

In a subsequent 2019 follow-up study of the same group (166), in which 48 patients with refractory pancreatic stenosis were included, after treatment with MPS, the stenosis was resolved in 83.3% (40/48) of patients. The 8 patients that were refractory to MPS treatment underwent a second MPS treatment with resolution of stenosis occurring in 3 patients. The 5 patients who did not show resolution of stenosis even after the second treatment refused surgery and continued to be treated endoscopically with a single plastic stent replacement annually or on-demand (167). During an average follow-up of 9.5 years, 74.4% (32/43) of treated patients that had resolution of stenosis remained asymptomatic. 25.6% (11/43) of the patients had a recurrence of pain after a mean time of 26.4 months and underwent endoscopic follow-up: in 3 of these patients a recurrence of pancreatic stenosis was evidenced requiring re-stenting while in the remaining 8 no stenosis was evidenced and the pancreatic duct was successfully drained after plugs extraction. No major complications were reported in all treatments performed.

The major drawback of this approach is the need for multiple sessions of endotherapy, which may affect patient compliance (168). Therefore, in patients with refractory pancreatic stenosis, the use of FCSEMS has been studied (169). In fact, specific FCSEMS have been developed that can be applied in pancreatic stenosis. These stents have a central portion with irregular cells that impart a different radial force that reduces their migration. In the prospective study with a long follow-up (170), this type of FCSEMS was placed in 15 patients, and after treatment, 90% of the patients remained asymptomatic after 3 years. Prior to insertion of the FCSEMS at the level of the pancreatic stenosis, removal of the previously placed plastic stent and a mechanical dilatation with an 8.5 F Soehendra dilator or, if the mechanical dilatation cannot be performed, a pneumatic dilatation with a 4 mm balloon should be performed. A 6 or 8 mm FCSEMS is then placed, depending on the diameter of the pancreatic duct upstream of the stenosis and varying in length depending on the stenosis. Finally, the FCSEMS is removed 6 months after placement.

In a recent meta-analysis of studies of patients with refractory pancreatic stenosis treated endoscopically (171), treatment with FCSEMS appears to have similar efficacy to treatment with MPS but has a higher risk of adverse events (38.6% vs 14.3%). The main adverse events reported with FCSEMS are risk of migration (12.8%), risk of bile duct occlusion (7.7%), and development of *de novo* pancreatic duct stenosis (up to 27%%). In contrast, the latter complication has never been reported in MPS treatment. The occurrence of abdominal pain such that the FCSEMS had to be removed was also reported in patients in whom a 10 mm FCSEMS, rather than an 8 mm FCSEMS, had been placed (172).

Pancreatic stenting is also an option to “by-pass” pancreatic stones obtaining ductal decompression and asses symptoms relief; pancreatic stenting in this setting is complex and need the integration with extracorporeal and intraductal lithotripsy.

## 6 Prophylactic pancreatic duct stenting

The risk of post-ERCP pancreatitis (PEP) increases if, inadvertently, the main pancreatic duct is opacified or a guidewire is inserted into it. A recent RCT (173), conducted in 167 patients undergoing ERCP for the first time in whom the pancreatic duct was inadvertently cannulated, compared placement of a 5 F pancreatic plastic stent with no stent placement. The study showed that prophylactic placement of a pancreatic plastic stent significantly reduced the risk of post-ERCP pancreatitis (12.6% vs. 25%). The most recent meta-analyses available in the literature (174, 175) also confirm this evidence. Therefore, guidelines (176) recommend that in case of inadvertent cannulation or opacification of the pancreatic duct, a prophylactic pancreatic stent should be placed. The use of a 3 or 5 cm 5 F plastic (177) pancreatic stent without an internal flange (178), but with a duodenal flange or pigtail is preferred to prevent intraductal migration of the stent (179). Spontaneous distal migration of the pancreatic stent should be confirmed by direct abdominal imaging after 7 to 15 days, and if the stent is still in place, endoscopic removal is necessary. Biodegradable stents are under evaluation for PEP prophylaxis avoiding the need for radiological control and eventual endoscopic procedure to remove the stent; lack of data and high cost limt the use of biodegradable bilio-pancreatic stents in clinical practice.

## 7 Stenting for failed extraction of biliary stones

If biliary lithiasis cannot be treated endoscopically because of a failure of available treatments and/or the patient’s clinical condition, temporary placement of a plastic stent is recommended (2).

A 10 F straight or double-pigtail plastic stent is usually placed, which allows passage of bile into the space created by the stent between the stone and that part of the biliary tract causing continuous friction of the stone, which aided by respiratory movement, reduces its size in 3-6 months in about half of the cases (180–182).

It is important to endoscopically re-evaluate the patient within 3 (183) to 6 months, because if left in place for longer, the plastic stent may promote the development of severe and even fatal cholangitis (184).

## 8 Stenting for post-sphincterotomy bleeding

In most cases, post-sphincterotomy bleeding resolves spontaneously (185). In cases of massive bleeding requiring urgent endoscopic treatment, placement of an FCSEMS is recommended when bleeding is refractory to standard hemostatic treatments (176). In retrospective studies, treatment with FCSEMS appears effective and safe (186, 187) and early placement of FCSEMS appears to reduce the incidence of recurrent bleeding (188). The optimal time for removal of FCSEMS after bleeding has stopped has not been established and is generally removed after a few days depending on the patient’s clinical condition. However, removal within 4 weeks is recommended to avoid the risk of complications related to “forgotten SEMS.”

## 9 Stenting during endoscopic papillectomy

Mucosal cautery during endoscopic papillectomy leads to edema of the pancreatic orifice resulting in duct occlusion and increased risk of developing post-ERCP pancreatitis (189).

There is only one RCT in the literature (190) aimed at evaluating the prophylaxis of post-papillectomy pancreatitis by placement of a pancreatic plastic stent. This RCT involved 19 patients who underwent endoscopic papillectomy and were randomized into two groups according to whether or not they underwent pancreatic plastic stent placement after ampullary adenoma resection. In patients who underwent pancreatic stent placement, a 3- or 5 cm-long single-flange 5 F straight pancreatic plastic stent was placed. The study reported a significantly higher incidence of post-procedure pancreatitis in the group without a pancreatic stent (33% vs 0%). A recent systematic review with pooled analysis (191) also showed that the incidence of post-procedure pancreatitis varied significantly in subgroup analysis based on whether or not a pancreatic stent was placed after endoscopic papillectomy.

Therefore, according to this evidence, the guidelines (189) recommend prophylactic placement of a plastic pancreatic stent after endoscopic papillectomy. The use of biodegradable stents may also be a promising indication (192).

Clearly, patients with pancreas divisum do not fall under this indication, thus it is important to define the patient’s anatomy by performing MRCP before the procedure. Biliary stenting after endoscopic papillectomy is not routinely indicated. In some cases, such as suspected endobiliary growth of the ampullary adenoma or bleeding, placement of a plastic biliary stent may also be considered.

## 10 Conclusions

**Figure 2:**
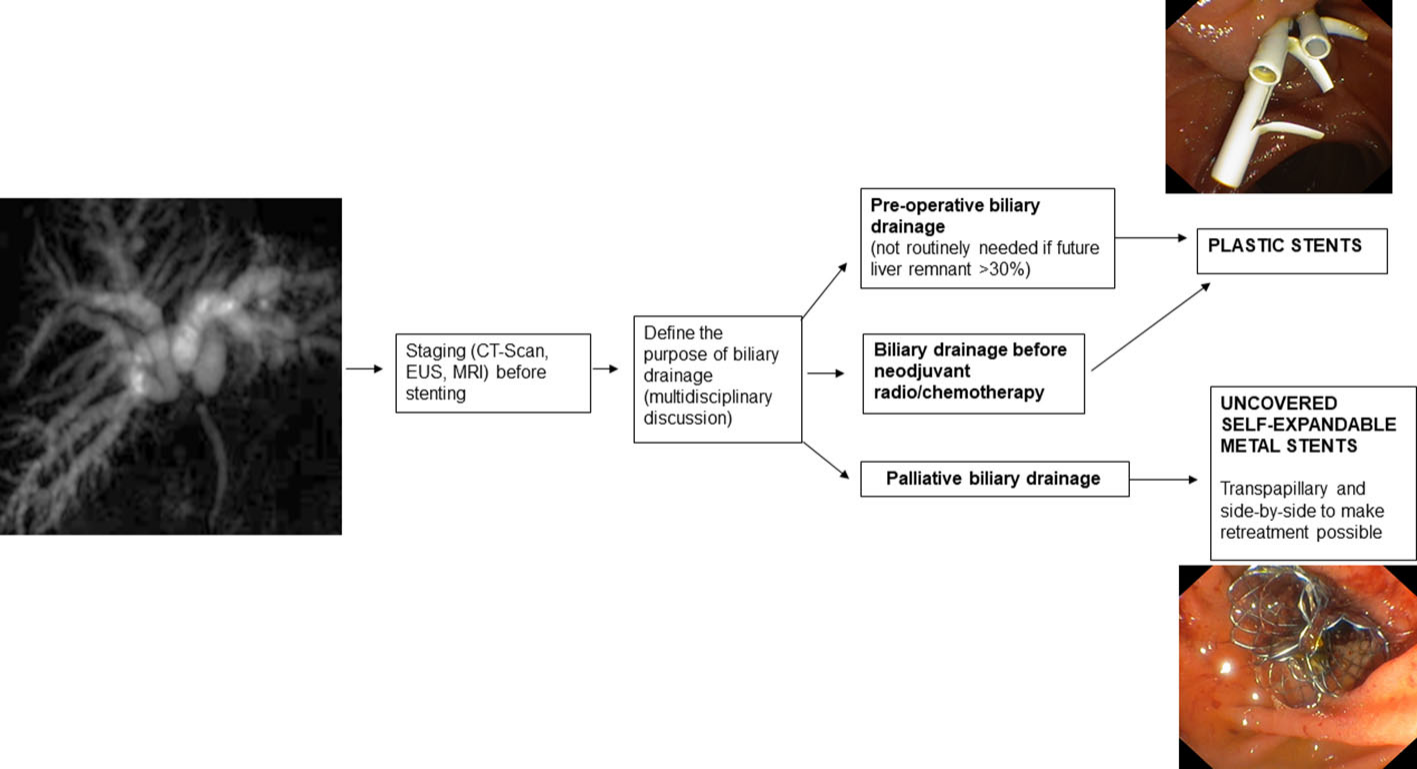
Malignant Hilar Strictures and choice of the stent.

**Figure 3:**
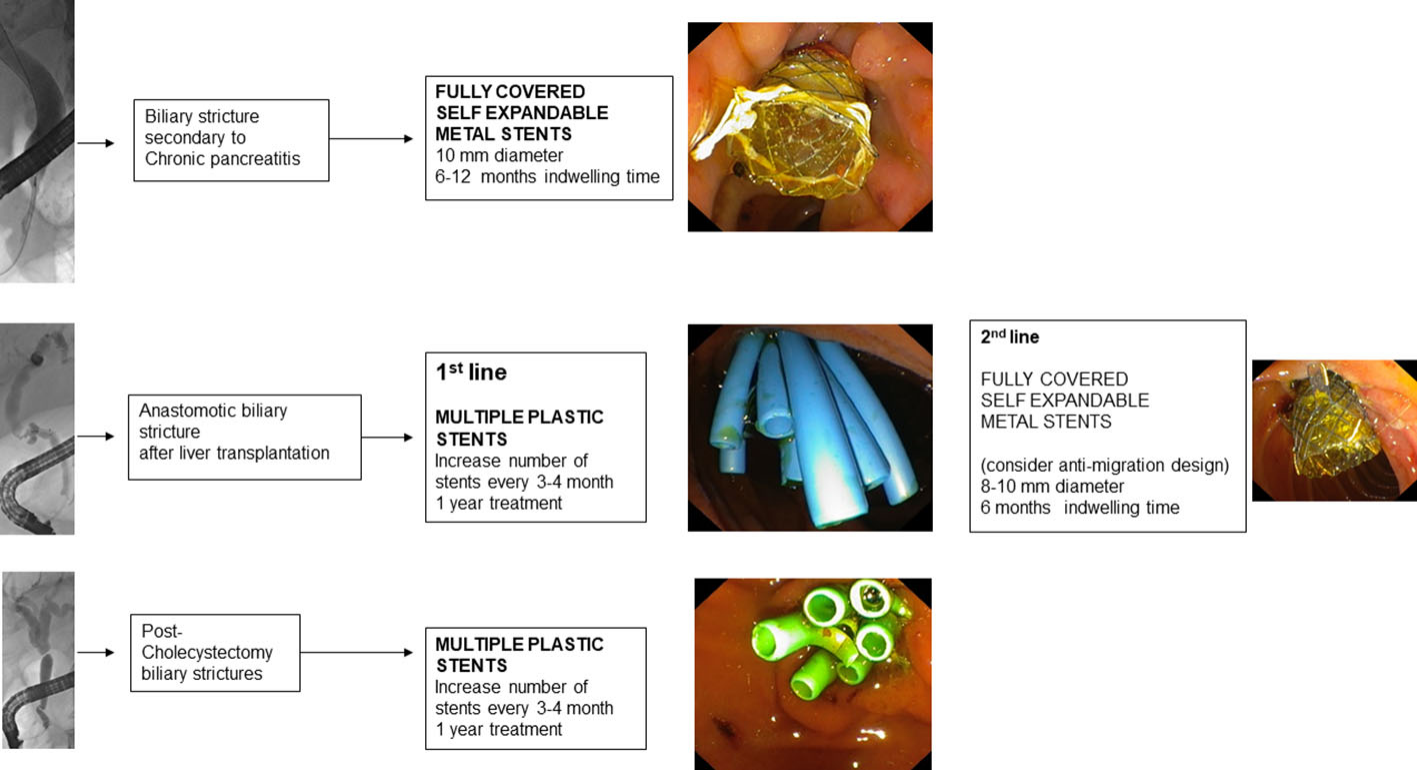
Benign biliary strictures and choice of the stent.

**Figure 4:**
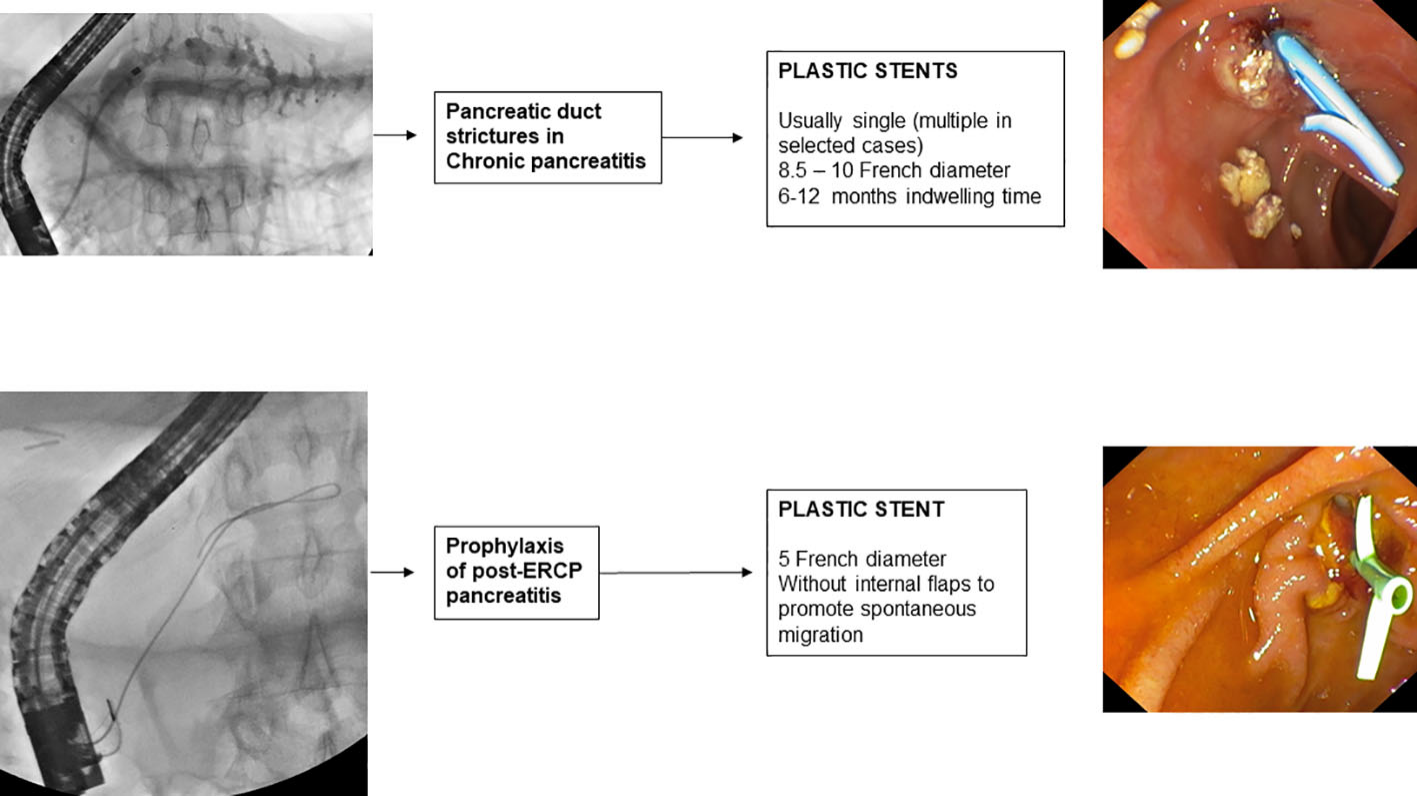
Pancreatic stenting and choice of the stent.

**Figure 5:**
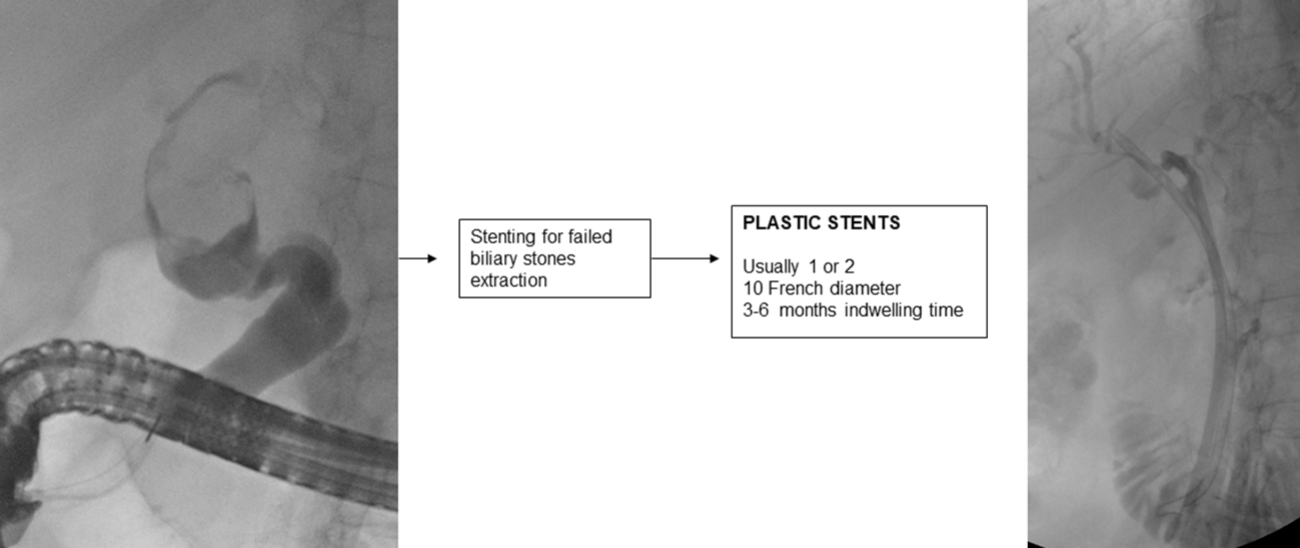
Biliary stenting for failed stones extraction.

**Figure 6:**
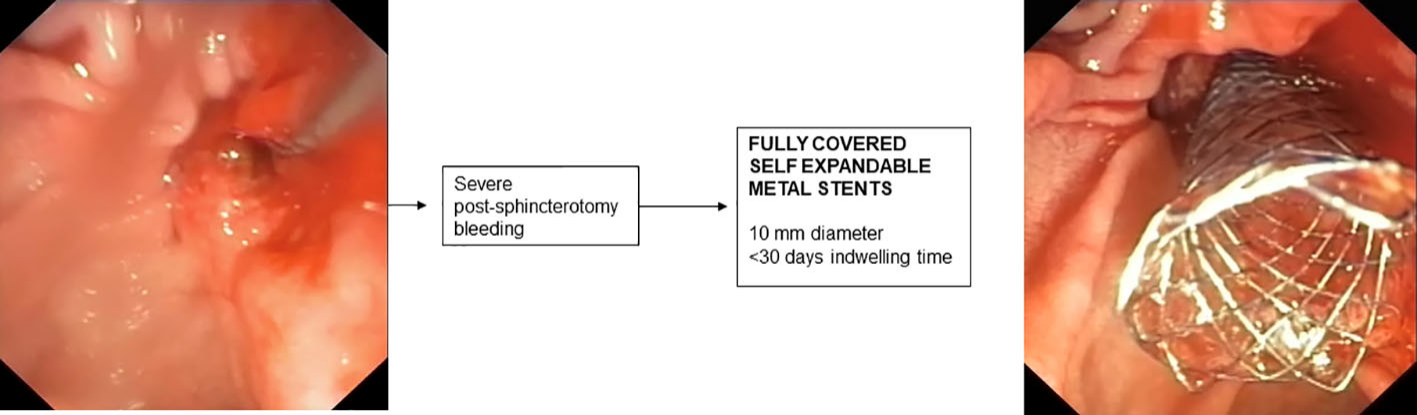
Fully covered metal stent for post-sphincterotomy bleeding.

**Figure 7:**
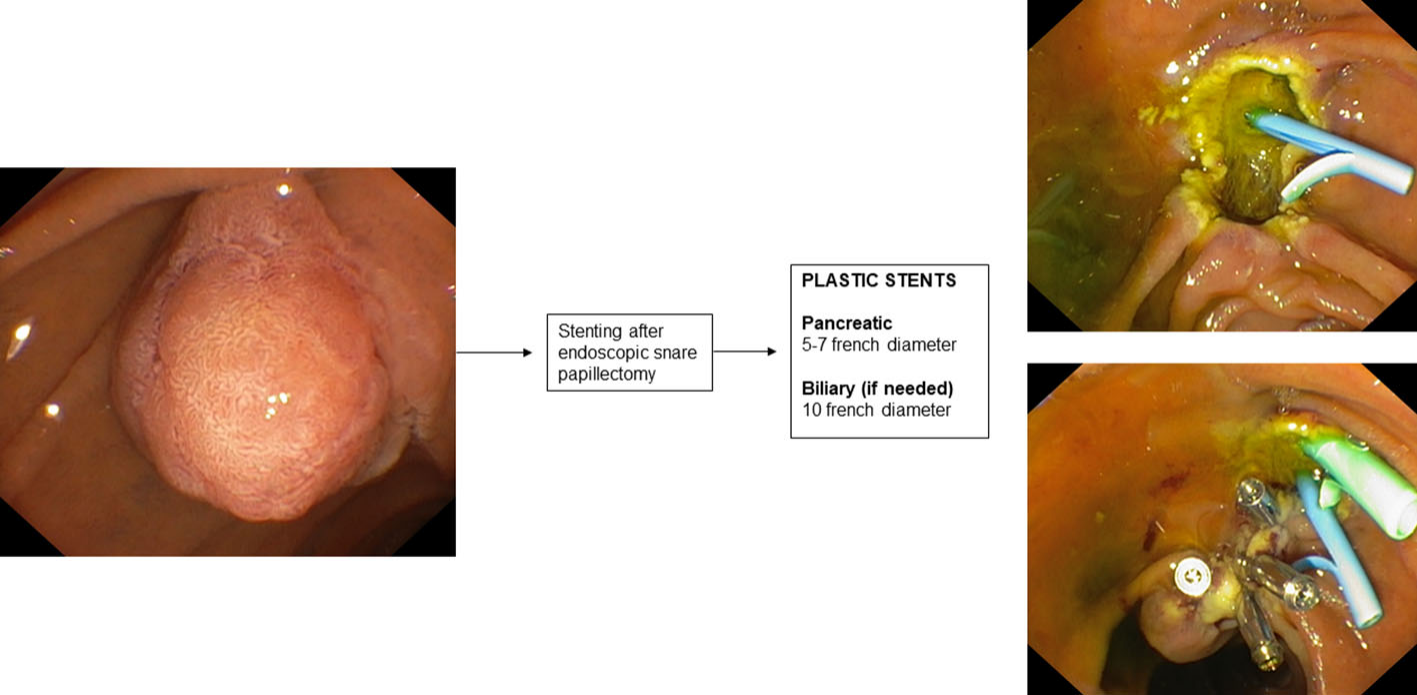
Stenting after endoscopic snare papillectomy.

Endoscopic bilio-pancreatic stenting is a first-line therapeutic option for palliation, pre-operative drainage and treatment of several bilio-pancreatic diseases. Correct choice of the stent is essential for a “tailored” treatment of such complex diseases, avoiding serious problems related to the so called “forgotten stent”, especially in benign diseases. **Figures 1**–**7** summarizes current indications and type of stents for an endoscopic approach to bilio-pancreatic strictures.

## Author contributions

AC, RL, and AT contributed to conception and design of the study. AC wrote the first draft of the manuscript. All authors contributed to the article and approved the submitted version.
